# A residual dense network assisted sparse view reconstruction for breast computed tomography

**DOI:** 10.1038/s41598-020-77923-0

**Published:** 2020-12-03

**Authors:** Zhiyang Fu, Hsin Wu Tseng, Srinivasan Vedantham, Andrew Karellas, Ali Bilgin

**Affiliations:** 1 Department of Medical Imaging, University of Arizona, Tucson, AZ USA; 2 Department of Electrical and Computer Engineering, University of Arizona, Tucson, AZ USA; 3 Department of Biomedical Engineering, University of Arizona, Tucson, AZ USA

**Keywords:** Computational science, Translational research

## Abstract

To develop and investigate a deep learning approach that uses sparse-view acquisition in dedicated breast computed tomography for radiation dose reduction, we propose a framework that combines 3D sparse-view cone-beam acquisition with a multi-slice residual dense network (MS-RDN) reconstruction. Projection datasets (300 views, full-scan) from 34 women were reconstructed using the FDK algorithm and served as reference. Sparse-view (100 views, full-scan) projection data were reconstructed using the FDK algorithm. The proposed MS-RDN uses the sparse-view and reference FDK reconstructions as input and label, respectively. Our MS-RDN evaluated with respect to fully sampled FDK reference yields superior performance, quantitatively and visually, compared to conventional compressed sensing methods and state-of-the-art deep learning based methods. The proposed deep learning driven framework can potentially enable low dose breast CT imaging.

## Introduction

Dedicated breast computed tomography (BCT) is an emerging, fully 3D, high-resolution (100–300 µm nearly isotropic voxels) imaging modality that does not employ physical compression of the breast. Compared to digital breast tomosynthesis^1^, BCT almost eliminates tissue superposition and does not suffer from limited-angle acquisition associated artifacts^2^ seen in digital breast tomosynthesis. A multi-reader, multi-case receiver operating characteristic (ROC) study employing 18 readers and 235 cases showed improved sensitivity of non-contrast diagnostic BCT over mammography-based diagnostic work-up^3^, leading to its regulatory approval for non-contrast diagnostic use. Non-contrast BCT can have a far greater role if its suitability for breast cancer screening is demonstrated. The radiation dose (mean glandular dose, MGD) from non-contrast diagnostic BCT, while similar to the MGD from mammography-based diagnostic workup, was approximately twice that of 2-view (standard) screening DM^4^. At radiation dose similar to mammography, a prior study using an early prototype showed improved visualization of masses and reduced visualization of microcalcifications with BCT compared to mammography^5^. Hence, the long-term goal is to reduce the radiation dose to be comparable to mammography screening, without loss of detection performance.

Radiation dose reduction in BCT to levels suitable for breast cancer screening can be achieved through improved hardware, acquisition strategies and advanced image reconstruction inclusive of post-processing techniques. In terms of hardware, photon-counting detectors^6,7^, low-noise, high-resolution, complementary metal oxide (CMOS) detectors^8,9^ and beam-shaping X-ray filters^10,11^ are being investigated. Acquisition strategies being investigated include helical scan^6^, laterally-shifted detector geometry^12,13^ short-scan^14^, and sparse-view acquisition^15^. Also, theoretical and empirical optimization of x-ray beam quality for acquiring projection data have been reported^16–19^.

In this study, we describe the potential of advanced image reconstruction employing deep learning techniques that can be used with existing BCT technology. This can lead to lower radiation dose and expedite its translation for breast cancer screening. This study is complementary to ongoing hardware-oriented research. Although, statistical iterative reconstruction^20–22^ and denoising techniques^23^ have been investigated for BCT, all BCT systems currently use Feldkamp–Davis–Kress (FDK) reconstruction^24^. Deep learning based image reconstruction has not been investigated in the context of BCT or for cone-beam CT; however, it has been explored for conventional multi-detector CT^25–28^. Jin et al.^25^ utilized the U-Net with residual learning and demonstrated the feasibility on parallel beam X-ray CT. A similar approach was independently proposed by Chen et al.^26^. The proposed residual encoder-decoder convolutional neural network (RED-CNN)^26^ was shown to be quantitatively outperforming the earlier version^29^ and the wavelet-domain CNN^30^.

Recently, advanced network architectures using residual blocks^31^ or dense blocks^32^ have shown improved performance compared to standard convolutional neural networks in computer vision applications^33,34^. In this work, we adopt a derived version of the residual dense network^33^ and investigate its potential for low-dose cone-beam BCT image reconstruction.

## Results

### Breast CT datasets

This retrospective study was conducted in accordance with relevant guidelines and institutional review-board (IRB) approved protocol (University of Arizona Human Subjects Protection Program, Protocol #1903470973). The study used de-identified projection datasets from 34 women assigned Breast Imaging-Reporting and Data System (BIRADS)^35^ diagnostic assessment category 4 or 5, who had previously participated in an IRB approved, Health Insurance Portability and Accountability Act (HIPAA)-compliant research study (ClinicalTrials.gov Identifier: NCT01090687). The study was conducted with informed consent from participants involved. This dataset was used in several prior studies^4,36–41^. All subjects underwent non-contrast dedicated breast CT exam of the ipsilateral breast using a clinical prototype flat-panel cone-beam breast CT system (Koning Corp., West Henrietta, NY). The scan parameters were: 49 kVp, 1.4 mm of Al 1st HVL, 8 ms pulse-width, 300 projection views, 360 degree full-scan acquisition, 12.6 mGy MGD, and 10 s scan time. The 300 view projection datasets were reconstructed using the FDK algorithm with 0.273 mm isotropic voxel pitch and matrix size $1024\times 1024$ in the transverse (coronal) plane. Sparse-view (100 views, full scan; 4.2 mGy MGD) projection data were retrospectively undersampled from the 300 view datasets and reconstructed with the FDK algorithm at the same voxel pitch. The longitudinal direction represents the slices. The 34 breast CT datasets were randomly split as follows: 20 for training (total of 8346 2D slices), 5 for validation (total of 1920 slices) and the remaining for testing (total of 4056 slices). The 9 test subjects were evenly divided into groups corresponding to small, medium, and large sized breasts, based on the number of slices in each case. The number of slices for the 9 test subjects were: 250, 315, 390, 426, 450, 462, 523, 600, and 640. The training dataset had diverse lesions (4 soft tissue lesions, 14 calcified lesions, and 2 soft tissue lesions with microcalcifications), BIRADS breast density categories (1, 6, 9, and 3 of categories a through d, respectively), and pathology (5 malignant, 2 hyperplasia, and the remaining benign).

### Impact of tissue of interest (TOI) selection

TOI selection was evaluated for the proposed multi-slice residual dense network (MS-RDN) and RED-CNN^26^. Test subject datasets were reconstructed by the single-slice networks with and without TOI selection. FDK reconstructions on the 300-view data (denoted as FDK300) were used as references across all the experiments. The performance was quantitatively evaluated with Normalized Mean Square Error (NMSE), bias, Peak Signal-to-Noise Ratio (PSNR), and Structural Similarity Index Metric (SSIM^42^). All four metrics significantly differed across all reconstructions (Wilks Lambda, $P<0.0001$). Table 1 panel (a) showed that TOI selection significantly improved all metrics for both RED-CNN and MS-RDN.

**Table 1:** Statistic analysis of the impact of TOI selection and multi-slice training for RED-CNN and MS-RDN architectures.

	RED-CNN	P-value	MS-RDN	P-value
**(a) Impact of TOI selection**				
NMSE	$+\,0.090$ dB	$<\,0.0001$	$+\,0.253$ dB	$<\,0.0001$
Bias	$-\,0.998\,\times 10^{-4}\,\text { cm}^{-1}$	$<\,0.0001$	$-\,2.099\times \, 10^{-4}\, \text { cm}^{-1}$	$<\,0.0001$
PSNR	$+\,0.090$ dB	$<0.0001$	$+\,0.253$ dB	$<\,0.0001$
SSIM	$+\,0.0009$	$<\,0.0001$	$+\,0.0011$	$<\,0.0001$
**(b) Impact of multi-slice training**				
NMSE	$+\,0.035$ dB	$<\,0.0001$	Not significant	0.211
Bias	$-\,0.411\,\times 10^{-4}\,\text { cm}^{-1}$	$<\,0.0001$	Not significant	0.234
PSNR	$+\,0.035$ dB	$<\,0.0001$	Not significant	0.211
SSIM	$+\,0.0004$	$<\,0.0001$	$+ \,0.0005$	$<\,0.0001$

The evaluation was performed on the entire testing breast dataset using NMSE, Bias, PSNR, and SSIM metrics.

(a) shows the performance improvement by including TOI selection on single-slice ($Z=1$) RED-CNN and MS-RDN.

(b) shows the performance difference between multi-slice ($Z=5$) and single-slice training for RED-CNN and MS-RDN, respectively. Please note that the values corresponding to NMSE and PSNR are identical since these quantities are related as shown in Eqs. (6) and (7).

### Impact of multi-slice training

**Figure 1:**
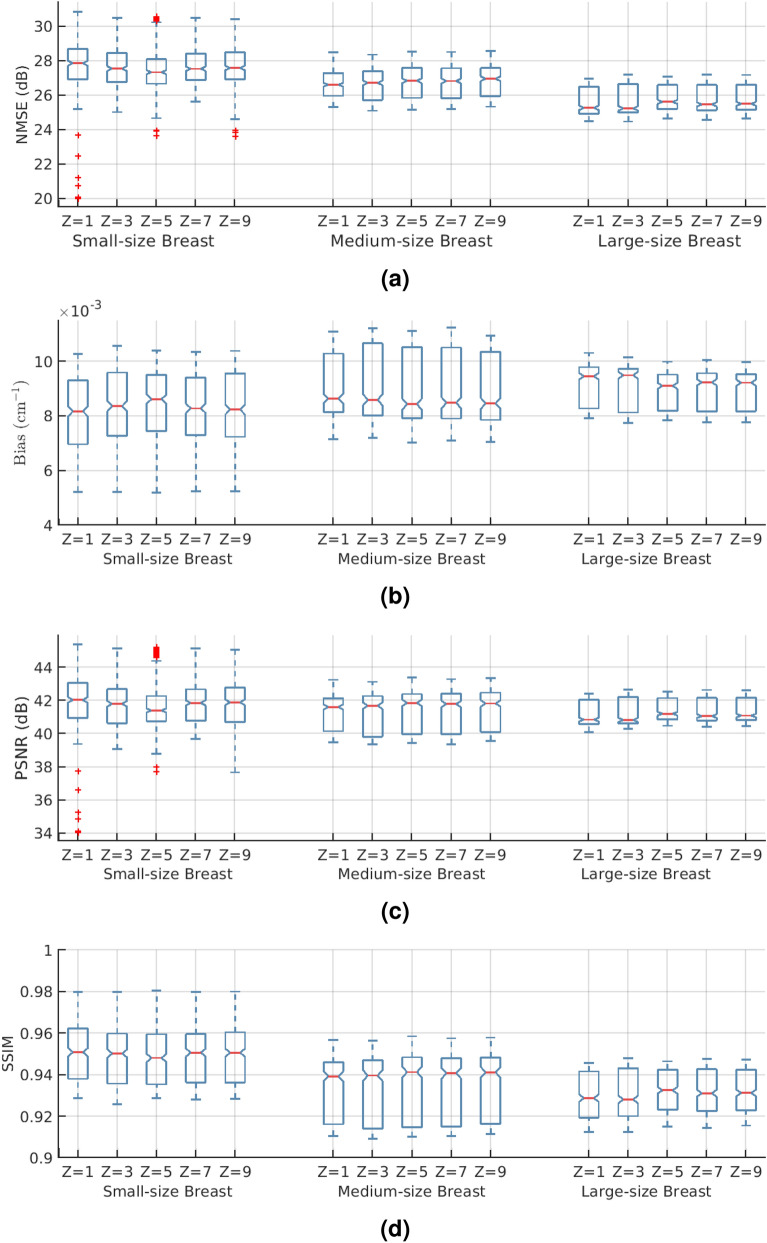
MS-RDN reconstructions with different number of adjacent slices ($Z=1, 3, 5, 7, 9$) are evaluated with (**a**) NMSE, (**b**) bias, (**c**) PSNR, and (**d**) SSIM for a range of breast sizes. Fully sampled FDK reconstructions are used as reference. These metrics computed along the longitudinal direction are presented using box plots. On each box, the central mark is the median, the top and bottom edges are the 25th and 75th percentiles, respectively. Outliers are denoted as red plus signs.

**Figure 2:**
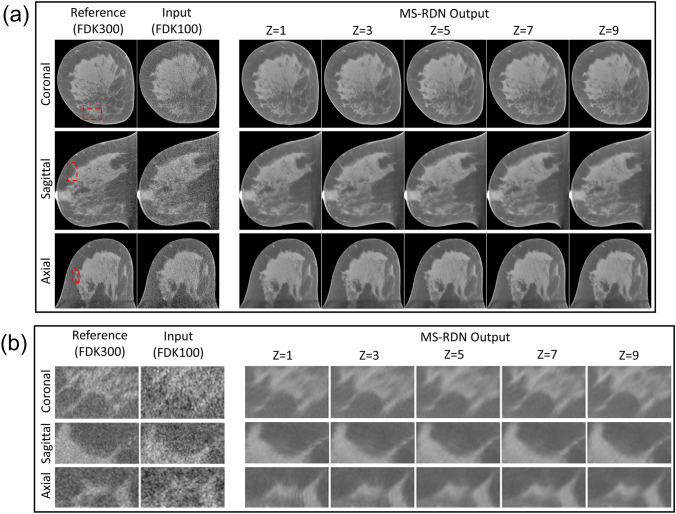
(**a**) A comparison of breast images reconstructed by MS-RDNs with different slice depth ($Z=1, 3, 5, 7, 9$) on retrospectively undersampled 100-view cone-beam data. The network inputs are obtained using FDK on the 100-view breast data, denoted as FDK100, and the references are obtained using FDK on the 300-view breast data, denoted as FDK300. The bounding boxes on the reference images indicate the ROIs enlarged in (**b**). Note that the sagittal and axial ROIs were rotated 90 degrees clockwise for presentation. The display window is $[0.15, 0.35]\, \text {cm}^{-1}$.

Over the entire test dataset, MS-RDN with $Z=1$ did not differ significantly from MS-RDN with $Z=5$ in terms of NMSE ($P=0.211$), bias ($P=0.234$), and PSNR ($P=0.211$) as shown in the panel (b) of Table 1. However, there was a significant improvement with MS-RDN5 compared to MS-RDNZ1 in SSIM (P<0.0001; mean improvement: 0.0005). For RED-CNN, multi-slice training significantly improved all metrics compared to single slice training. The boxplots in Fig. 1 show independent evaluations for small-size, medium-size, and large-size breasts. Figure 1a shows relatively consistent NMSE performance from small-size breasts to large-size breasts. Similar observation of robust performance can be made for the bias, PSNR, and SSIM boxplots shown in Fig. 1b–d, respectively. The quantitative performances of MS-RDN and RED-CNN with multi-slice training were breast size dependent with smaller improvements, or degradation, for smaller breasts than for medium and large breasts. For the medium-size and large-size breasts, MS-RDN with Z = 5 (MS-RDNZ5) achieved the best performance for all metrics. For small-size breasts, the single-slice MS-RDN (MS-RDNZ1) appeared to perform better than multi-slice networks. The lower cone-angle of small-size breasts could reduce longitudinal correlation for the multi-slice networks to exploit, and the under-representation of small-size breasts (approximately 16% of slices) in the training dataset may be contributing factors to the above observation. Studies into these aspects will be pursued in future with the availability of larger datasets. Figure 2a shows the (medium-size) breast images reconstructed by FDK and MS-RDNs with varying slice depths on the retrospectively undersampled 100-view data together with the reference image obtained using FDK on the 300-view data. Figure 2b shows the zoomed-in views corresponding to the red bounding boxes indicated in Fig. 2a. Note that the sagittal and axial ROIs were rotated 90 degrees clockwise for display. Compared to the reference images, all MS-RDN outputs appear less noisy. It is worth noting that the Venetian blind artifacts appear in the longitudinal reconstructions of MS-RDN with single slice training. As the slice depth increases, these artifacts are suppressed but the glandular tissues become blurred gradually. Importantly, multi-slice training eliminates longitudinal artifacts and enhances the reconstructions as well. On the other hand, MS-RDN with large slice depths increases computational complexity in training and testing without gaining substantial performance. Hence, we opted to train MS-RDN with 5 adjacent slices in the following experiments as a balance between performance and complexity.

### Comparison with RED-CNN

**Figure 3:**
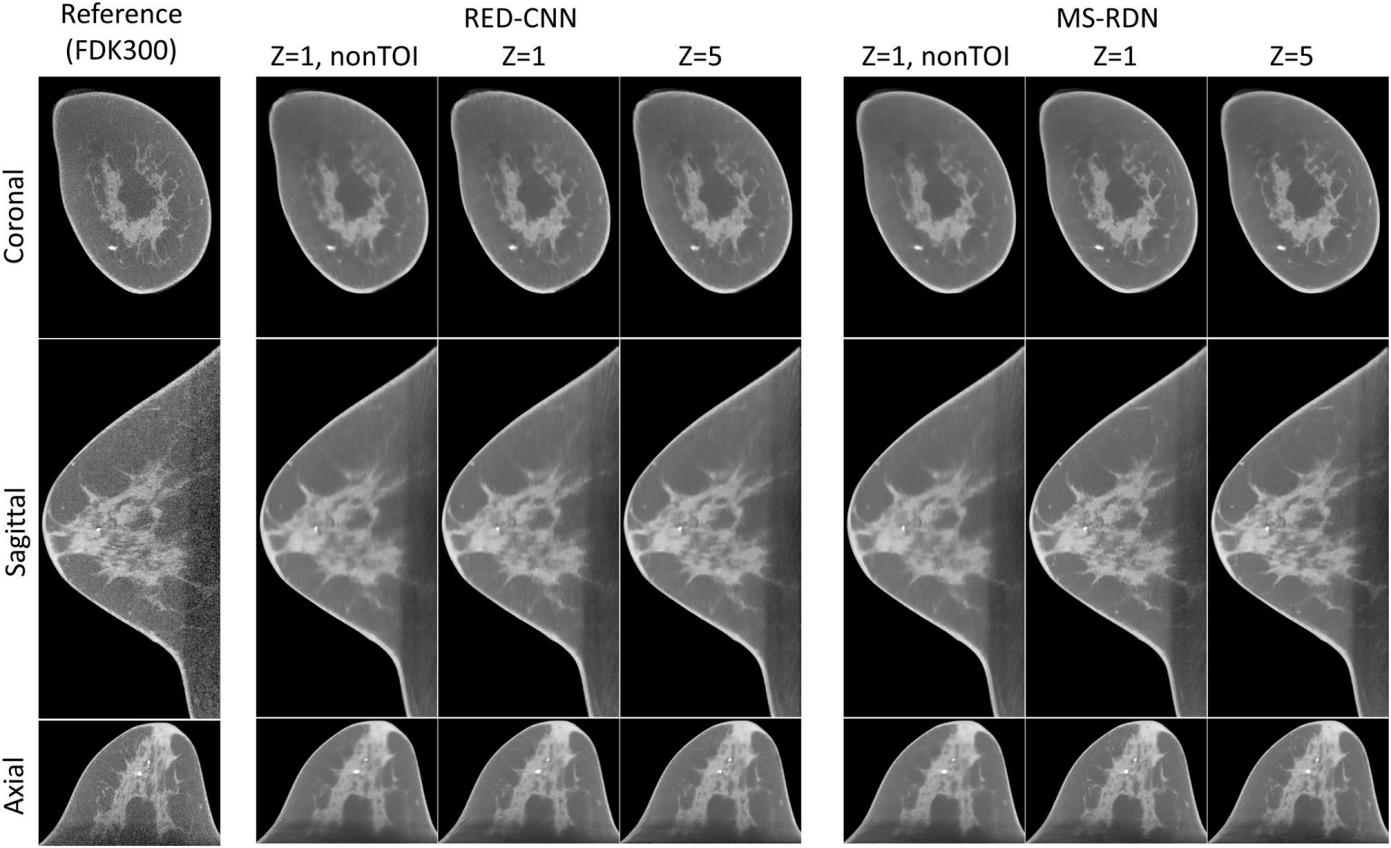
Comparisons to the residual encoder–decoder convolutional neural network (RED-CNN). The proposed MS-RDN was compared with RED-CNN in three sets of configurations: single slice training without TOI oriented patch extraction ($Z=1$, nonTOI), single slice training ($Z=1$), and multi-slice training ($Z=5$). Breast images of the test subject were reconstructed by these RED-CNNs and MS-RDNs using the retrospectively undersampled 100-view data. The reference images were obtained using FDK on the 300-view data. The display window is $[0.15,0.35]\, \text {cm}^{-1}$.

**Figure 4:**
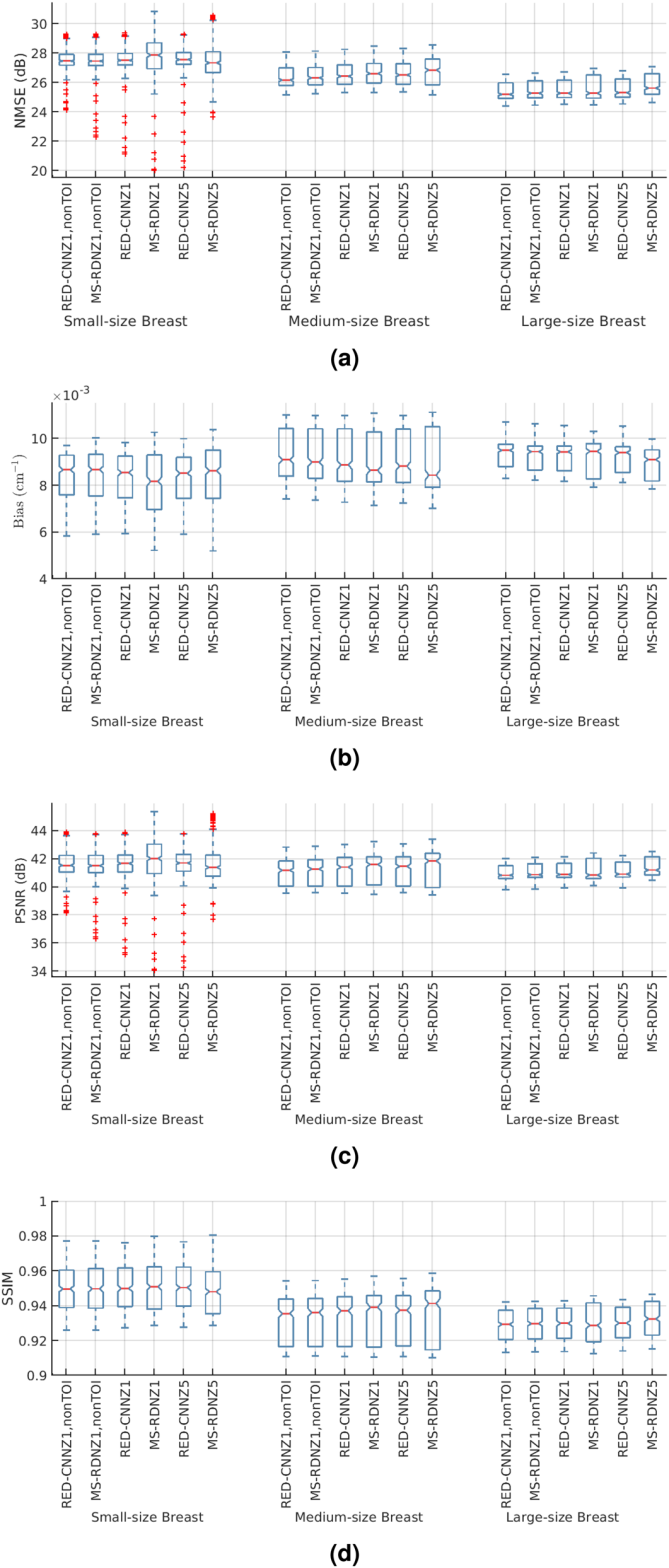
The boxplots of (**a**) NMSE, (**b**) bias, (**c**) PSNR, and (**d**) SSIM for the reconstructions obtained using RED-CNN and MS-RDN with the following configurations: single slice training without TOI oriented patch extraction ($Z=1$, nonTOI), single slice training ($Z=1$), and multi-slice training ($Z=5$). For example, “MS-RDNZ1” represents MS-RDN with single slice training. On each box, the central mark is the median, the top and bottom are the 25th and 75th percentiles respectively. Outliers are denoted as red plus signs. Note that, in each breast-size group, MS-RDN and RED-CNN with the same configurations are placed next to each other for comparison.

Our MS-RDN was compared with RED-CNN in three sets of network configurations: single slice training without TOI selection ($Z=1$, nonTOI), single slice training ($Z=1$), and multi-slice training ($Z=5$). Figure 3 shows the breast images (small-size) reconstructed by RED-CNN and MS-RDN on the retrospectively undersampled 100-view data together with the reference image obtained using FDK on the 300-view data. Overall, MS-RDNs preserved high-frequency features such as edges and textures better than their RED-CNN counterparts. In addition, the aforementioned Venetian blind artifacts are also presented in the non-transverse images obtained using RED-CNN with single slice training. Figure 4 shows the boxplots of (a) NMSE, (b) bias, (c) PSNR, and (d) SSIM for the RED-CNN and MS-RDN reconstructions of various-size breasts. For small-size breasts, MS-RDN with single slice training ($Z=1$) attained the best NMSE and bias performance. For medium-size and large-size breasts, it can also be observed that TOI selection and multi-slice training improve performance of MS-RDN independently. Table 2 shows that MS-RDN outperforms RED-CNN significantly in all configurations.

**Table 2:** Statistical analysis of MS-RDN and RED-CNN reconstructions using generalized linear models.

	NMSE (dB)	Bias ($\text { cm}^{-1}$)	PSNR (dB)	SSIM
Single slice training, non-TOI	$+\,0.034$	$-\,0.399\,\times 10^{-4}$	$+\,0.034$	$+\,0.0003$
Single slice training	$+\,0.197$	$-\,1.501\,\times 10^{-4}$	$+\,0.197$	$+\,0.0006$
Multi-slice training ($Z=5$)	$+\,0.144$	$-\,0.975\,\times 10^{-4}$	$+\,0.144$	$+\,0.0007$

The table reports the performance gained by MS-RDN over RED-CNN for three different configurations and four quantitative metrics.

All improvements are significant with $P<0.0001$. Please note that the values corresponding to NMSE and PSNR are identical since these quantities are related as shown in Eqs. (6) and (7).

### Comparison with the fast, iterative, TV-regularized, statistical reconstruction technique (FIRST^22^)

**Figure 5:**
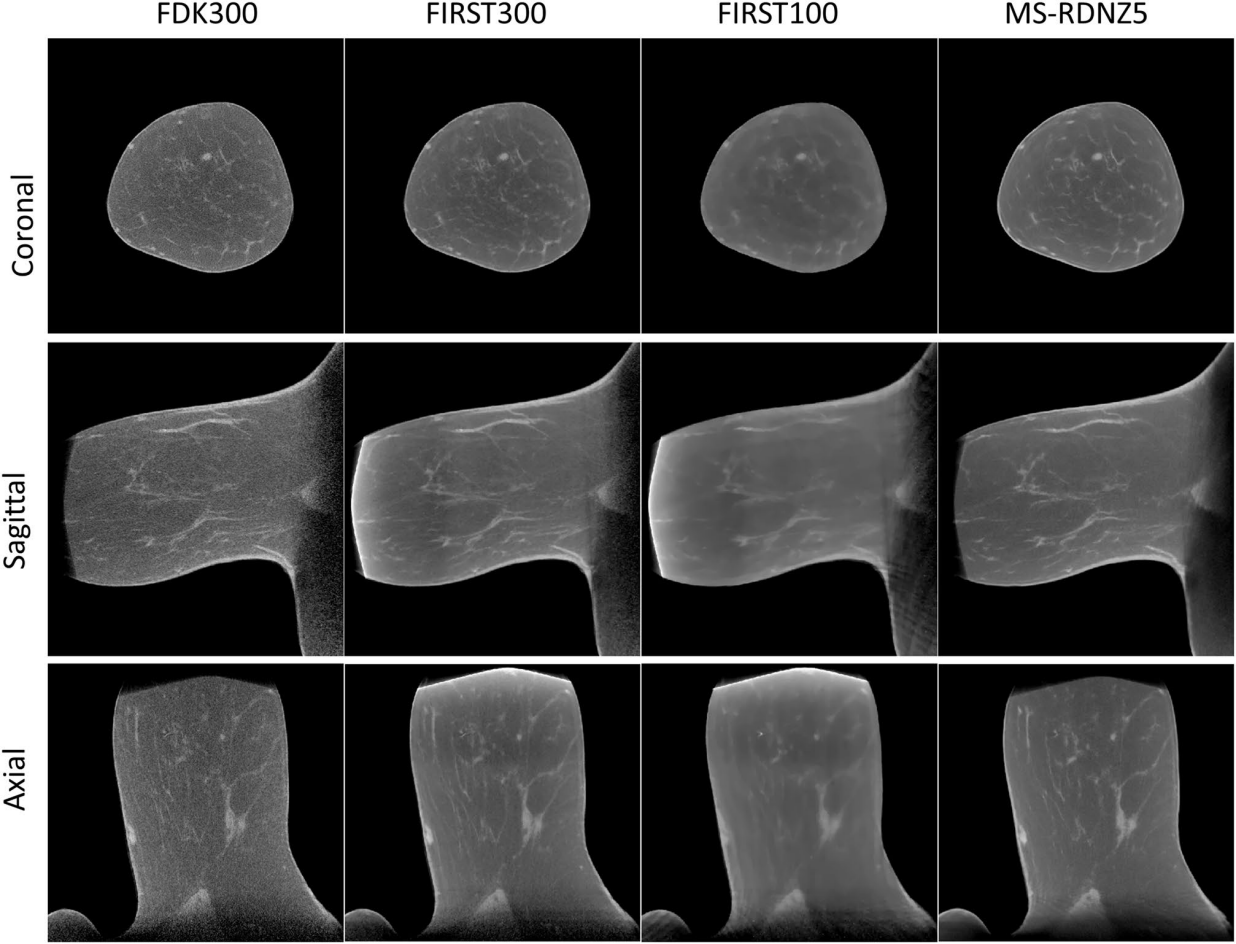
A comparison to the FIRST algorithm. Breast reference images, FDK300 and FIRST300, are obtained using FDK and FIRST algorithms on the 300-view data respectively. Similarly, FIRST100 represents FIRST reconstructions on the retrospectively undersampled 100-view data. On the same undersampled data, breast images were reconstructed using MS-RDN with multi-slice training ($Z=5$), indicated as MS-RDNZ5. The display window is $[0.15, 0.35]\, \text {cm}^{-1}$.

Figure 5 illustrates the breast (large-size) reference images reconstructed by FDK and FIRST using the 300-view data as well as the reconstructions obtained using FIRST and MS-RDNZ5 on the 100-view data. Compared to the 300-view FDK reconstructions (FDK300), the 300-view FIRST reconstructions (FIRST300) suppress the noise and preserve breast tissue structures in fine scale. However, the FIRST reconstructions with the 100-view data (FIRST100) exhibits blurred structures/textures and increased streak artifacts. In contrast, MS-RDNZ5 with 100-view data is able to remove the streaks as well as suppress the noise. In Table 3, the performance of FIRST and MS-RDNZ5 are evaluated with NMSE, bias, PSNR, and SSIM using 300-view FDK and 300-view FIRST reconstructions as references, respectively. For all these metrics, MS-RDNZ5 outperforms FIRST considerably. It is noteworthy that these metrics are improved by a large margin (roughly 5–8 dB NMSE increase, 4–6 $\times 10^{-3}$cm$^{-1}$ bias decrease, 5–8 dB PSNR increase, and 0.04–0.07 SSIM increase) when FIRST300 images rather than FDK300 reconstructions are used as references.

**Table 3:** Quantitative analysis of the proposed method (MS-RDNZ5) and the FIRST algorithm.

Metrics	FDK300 reference		FIRST300 reference	
	FIRST100	MS-RDNZ5	FIRST100	MS-RDNZ5
**NMSE (dB)**				
S	27.19 (1.03)	**27.50** (1.03)	32.67 (1.01)	**32.99** (1.17)
M	24.85 (0.19)	**25.67** (0.31)	29.15 (0.36)	**31.45** (1.53)
L	26.11 (0.38)	**26.78** (0.29)	34.93 (0.52)	**35.68** (0.71)
**Bias** ($10^{-3}$ **cm**$^{-1}$)				
S	9.08 (1.24)	**8.60** (1.65)	4.55 (0.71)	**4.12** (0.81)
M	11.80 (0.21)	**10.69** (0.29)	6.98 (0.26)	**5.19** (0.79)
L	8.68 (0.34)	**8.07** (0.19)	2.92 (0.19)	**2.75** (0.26)
**PSNR (dB)**				
S	41.17 (1.17)	**41.42** (1.37)	46.77 (1.09)	**47.00** (1.28)
M	38.95 (0.16)	**39.80** (0.25)	43.23 (0.31)	**45.56** (1.43)
L	41.62 (0.32)	**42.28** (0.21)	50.45 (0.53)	**51.22** (0.73)
**SSIM**				
S	0.941 (0.020)	**0.946** (0.021)	0.988 (0.004)	**0.989** (0.003)
M	0.893 (0.004)	**0.914** (0.003)	0.964 (0.003)	**0.985** (0.002)
L	0.938 (0.003)	**0.944** (0.002)	**0.994** (0.001)	**0.994** (0.001)

One small-size breast (S), one medium-size breast (M), and one large-size breast (L) were selected for testing, respectively. The suffixes “100” and “300” denote the number of projections in the data. The MS-RDNZ5 network was always trained using FDK100 as input and FDK300 as label. However, either FDK300 or FIRST300 were used as the reference when computing the quality metrics, as indicated by the column labels “FDK300 Reference” and “FIRST300 Reference”, respectively.

Median and interquartile range in the bracket are shown.

Bolded values indicate better performance in pairwise comparison.

### Outlier inspection

**Figure 6:**
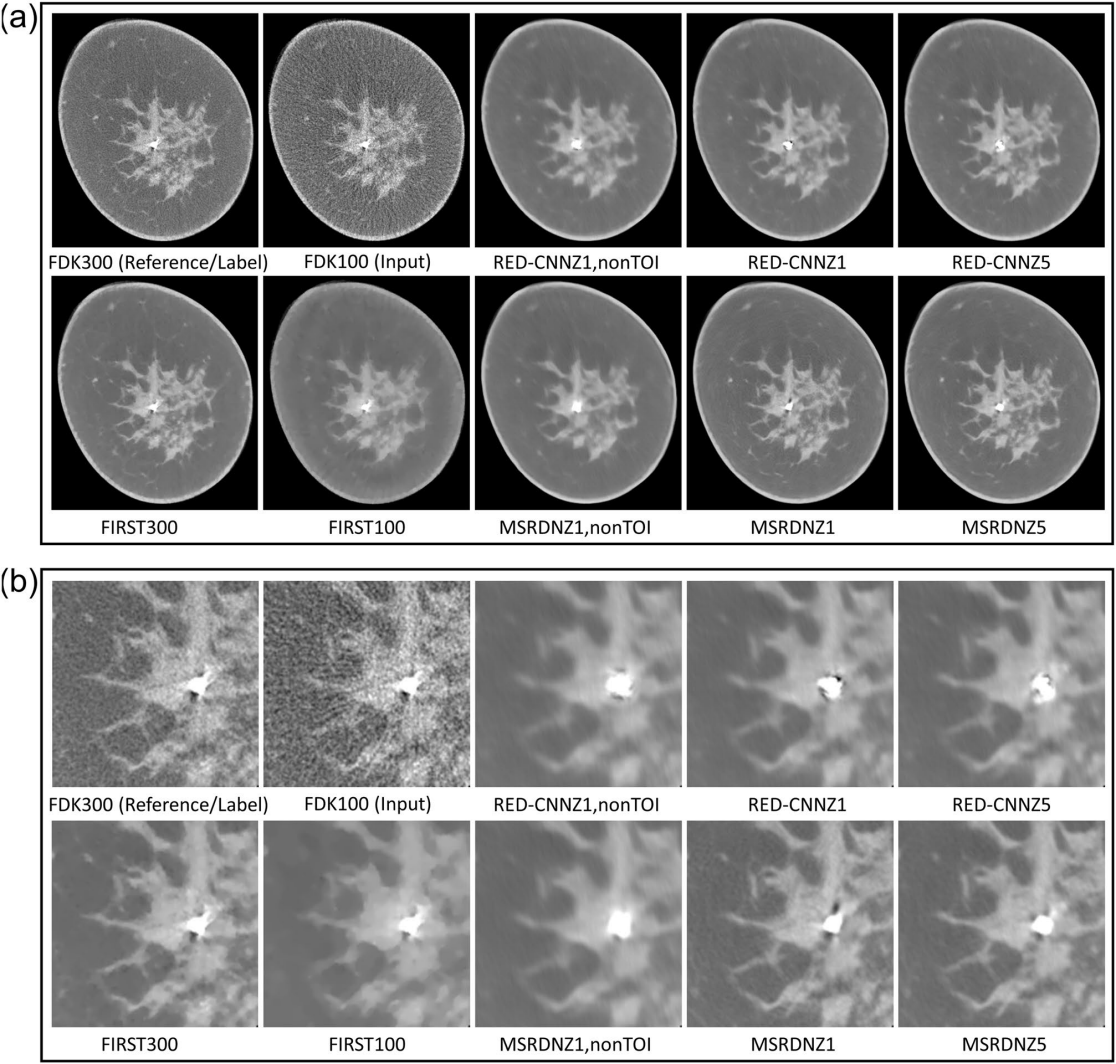
Reconstructions of the slice that yields the worst NMSE performance for MS-RDNZ5 in Fig. 4a. Reconstructions from all investigated methods are shown in (**a**). The zoomed regions of the central part of the breast tissue with a calcification are shown in (**b**). The display window is $[0.15, 0.35]\, \text {cm}^{-1}$.

The slice with the worst NMSE for MS-RDNZ5 was identified in Fig. 4. This slice was from a small heterogeneously dense breast (BI-RADS density category c). Figure 6 shows the reconstructions obtained using the investigated methods for this slice. A hyper-intense signal, corresponding to a calcification, is located near the center of the breast, which was biopsied subsequent to breast CT. Pathology indicated a benign finding—fibrosis with calcification. It is interesting to note that this calcification is not reconstructed well by any of the deep-learning techniques in terms of the shape, whereas the iterative reconstruction captures the shape better. However, there is loss of detail and texture in other regions, such as the edges between adipose and fibroglandular tissues, with the iterative reconstruction.

## Discussion

In this study, we presented a deep learning (DL) based reconstruction framework for 3D sparse-view breast CT. In reference to full view FDK reconstructions, the proposed framework yields image quality superior to compressed sensing techniques such as FIRST while requiring comparable reconstruction times. In this study, the reconstructed FOV was relatively large (280 mm $\times$ 280 mm or 1024 pixel $\times$ 1024 pixel) to accommodate breasts with large diameter at the chest-wall^36^, which leads to large fraction of background in some of the datasets. Thus, we adopted a tissue of interest oriented patch extraction strategy, termed TOI selection, during the network training to enforce learning on the breast tissue region rather than the irrelevant background regions. Importantly, patches that contain less than 50% background pixels were also included in training to ensure the recovery of breast anatomy boundary. This TOI selection alone enhanced the sharpness of breast textures and achieved improved NMSE and bias compared to random patch extraction.

This work used multi-slice training as a compromise between 2D and 3D network training. We demonstrated that multi-slice training is effective in exploiting the correlations between adjacent slices. Most importantly, it eliminated the Venetian blind artifacts in images obtained using single slice training. However, we also noticed that the performance gained with increased slice depth of MS-RDN saturates at small number of slice depth. This suggests the longitudinal correlation is largely local. One future extension to the current work could be assembling three networks trained in the axial, coronal, and sagittal planes respectively. The ensemble of three 3D networks explores local similarities along all three orientations similar to what a 3D network does but it would still require much less GPU memory and training data.

Our DL-based framework uses residual dense blocks^33,43^ as the backbone of the network. It has been shown that such combination of residual connections^31^ and densely connected structures^32^ improved network parameter efficiency and reconstruction accuracy in single image super resolution problems^33,43^. Our MS-RDN was comprehensively compared with the residual learning based RED-CNN and showed superior reconstruction quality of breast CT images. While this study demonstrated promise in the task of sparse-view breast CT reconstruction, it has several limitations. The reference FDK reconstruction exhibits higher noise than multi-detector CT used for imaging other organs, due to the hardware limitations and radiation dose constraints. Our MS-RDN reconstructions looked (perceptually) more similar to the FIRST approaches in terms of signal-to-noise ratio. Recent studies^44–46^ suggest that pixel-wise losses, such as $\ell _1$ or $\ell _2$ loss, are prone to overly smoothing image structures. In contrast, adversarial training^47,48^, perceptual loss^49^, as well as texture matching loss^50^ are proven to preserve high frequency image content and improve the perceptual quality. However, it should be noted that these techniques may hallucinate high frequency textures^44^, which makes them less appealing for medical applications. In breast CT imaging, hallucinated high frequency texture may mimic microcalcifications. Nevertheless, the impact of alternative loss functions in dedicated breast CT needs to be investigated and can be an extension of the current work.

We also investigated the possible failure cases for the proposed deep learning technique. For the example shown in Fig. 6, we found out that both MS-RDN and RED-CNN (irrespective of their configurations) produced poor reconstructions of the shape of a calcification. Note that the calcification is a minor class compared to the fibroglandular or adipose tissues in the training dataset. Unlike the iterative compressed sensing method, which includes data consistency and model based priors, the proposed method learns from training samples. Hence, the network may not learn the characteristics of tissues that are scarcely represented in the training data. It would be interesting to develop deep learning techniques that can yield improved reconstructions of such calcifications in future works.

## Methods

### Projection acquisition and three-dimensional image reconstruction

In 3D cone-beam BCT, multi-projection data ${\mathbf {P}}\in {{\mathbb {R}}^{N_d \times N_p}}$ were acquired in a complete circular trajectory composed of $N_p$ projections using a two-dimensional (2D) X-ray area detector consisting of $N_d$ pixels. From the cone-beam projections $\mathbf {P}$, an estimate of the underlying image volume ${\mathbf {V}} \in {{\mathbb {R}}^{N_x \times N_y \times N_z }}$ was reconstructed using the conventional analytical FDK algorithm^24^. The reconstruction process can be expressed using the following equation

1 $$\begin{aligned} {\mathbf {V = F(P)}}, \end{aligned}$$

where $\mathbf {F}$ denotes the FDK reconstruction operator interpolated by voxel-driven approach^51,52^. Reconstructed volumes are assumed to have isotropic voxel resolution as the voxel sizes are principally determined by size of the imaging detectors. However, the spatial resolution can be location-dependent and anisotropic due to reduced sampling at the periphery of the field of view within a transverse slice and due to geometric distortions arising from cone-beam geometry (commonly referred to as cone-beam artifacts) as the acquisition does not satisfy data-completeness requirement^53,54^ with the exception of the central transverse slices.

To reduce radiation dose, a common way is to uniformly reduce the number of projections without compromising the full angular coverage^55–57^. This sparse-view projection data was obtained by retrospectively undersampling the full-view projection data $\mathbf {P}$ using

2 $$\begin{aligned} {{\mathbf {P}}_{u}}= {\mathbf {P}}[1:1:N_d,1:u:N_p], \end{aligned}$$

where ${\mathbf {P}}_u \in {\mathbb {R}}^{N_d \times \lfloor \frac{N_p}{u} \rfloor }$ represents the sparse-view projection data, *u* denotes the undersampling factor, and the notation *i*: *j*: *k* in Eq. (1) denotes regularly spaced sampling between indices *i* and *k* using *j* as the increment. Similarly, an estimate of the image volume ${\mathbf {V}}_u$ was reconstructed from the sparse-view data ${\mathbf {P}}_u$ using the FDK algorithm, that is

3 $$\begin{aligned} {\mathbf {V}}_u= {\mathbf {F}}({\mathbf {P}}_u). \end{aligned}$$

It should be noted that the reconstructed image volume ${\mathbf {V}}_u$ typically exhibits streaking artifacts due to undersampling.

### Deep neural network reconstruction

Earlier studies on abdominal contrast-enhanced CT^58^ and optoacoustic tomography^59^ showed promising performance of deep neural network reconstruction with sparse data. The goal of this work is to combine sparse-view data acquisition with deep neural network reconstruction to reduce undersampling artifacts. A deep neural network $\mathbf {D(w,\cdot )}$ can be utilized to recover $\mathbf {V}$ from ${\mathbf {V}}_u$, where $\mathbf {w}$ are the weights of $\mathbf {D}$. In supervised learning, $\mathbf {w}$ are optimized by minimizing a pre-defined loss function $\mathbf {L(\cdot )}$, namely,

4 $$\begin{aligned} {\hat{\mathbf {{w}}}} = \underset{\mathbf {w}}{\arg \max }\,\, {\mathbf {L}}({\mathbf {D}}({\mathbf {w}}, {\mathbf {V}}_u ), {\mathbf {V}}) \end{aligned}$$

over a training dataset.

Our proposed framework uses supervised training where the inputs and targets of the network are obtained using Eqs. (1) and (3), respectively. While it may be ideal to process the entire volume using a 3D neural network, there are practical constraints associated with 3D networks^60–65^. Conventional denoising methods for 3D CT images based on non-local means^66^ or block matching filter^67^ showed that a multi-slice approach is able to leverage inter-slice spatial dependencies with small growth in computational complexity. Hence, we jointly reconstruct $Z\in \mathbb {Z^+}$ adjacent slices as a compromise between 2D and 3D processing.

**Figure 7:**
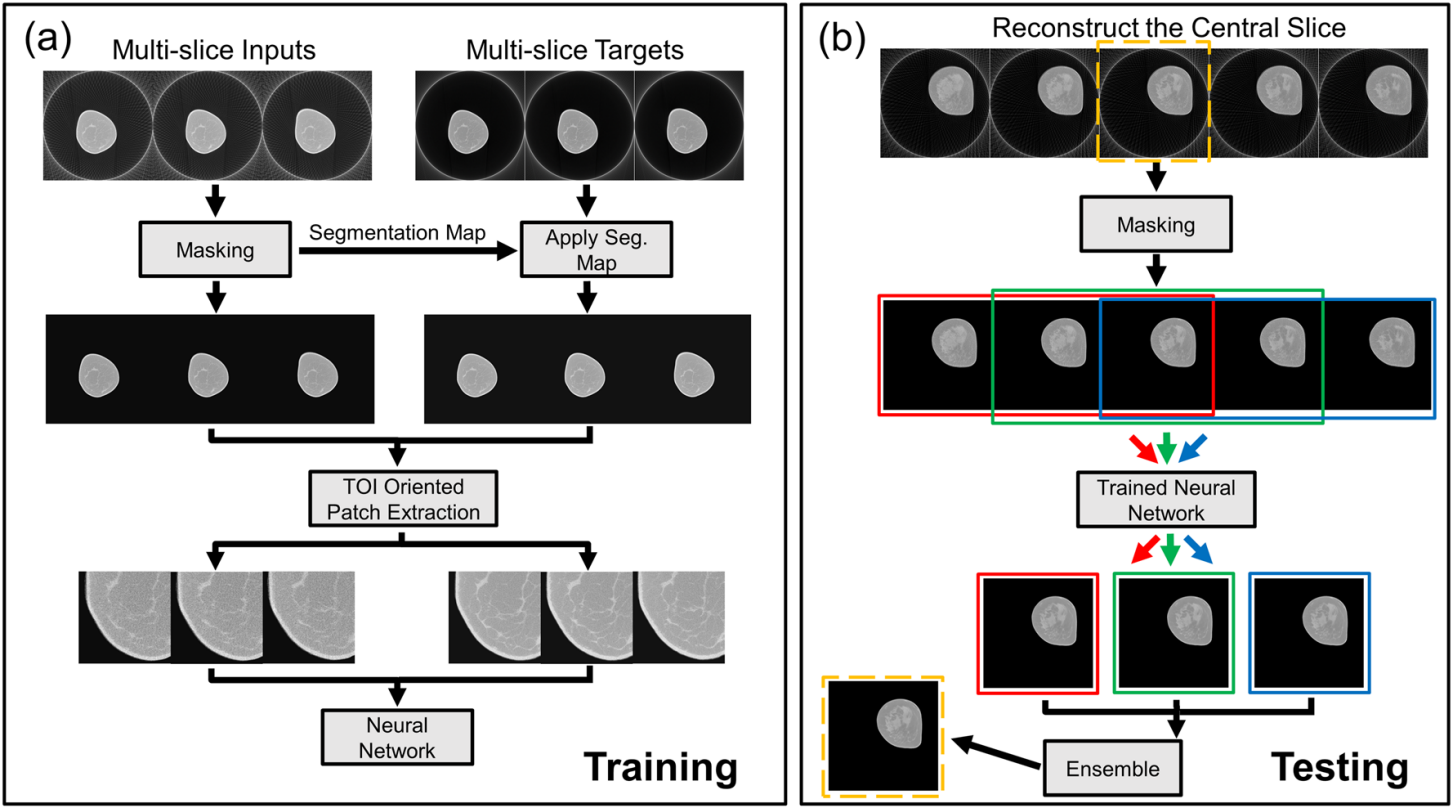
Network multi-slice (**a**) training and (**b**) testing framework. Training with three slices is shown as an example. (**a**) Multi-slice inputs reconstructed from sparse projection data is processed with the masking procedure described in Fig. 8. The generated segmentation maps are shared with multi-slice targets reconstructed from full projection data. Patches are extracted as training samples only when they contain more than 50% foreground pixels based on the generated masks, termed tissue of interest (TOI) oriented. (**b**) Five consecutive testing slices are used to reconstruct the central slice, indicated by the yellow bounding box. Three sets of multi-slice inputs, where the target slice has different slice context, are independently processed by the same trained network. Only the target slices are retained and aggregated to obtain the final reconstruction of the target slice.

**Figure 8:**
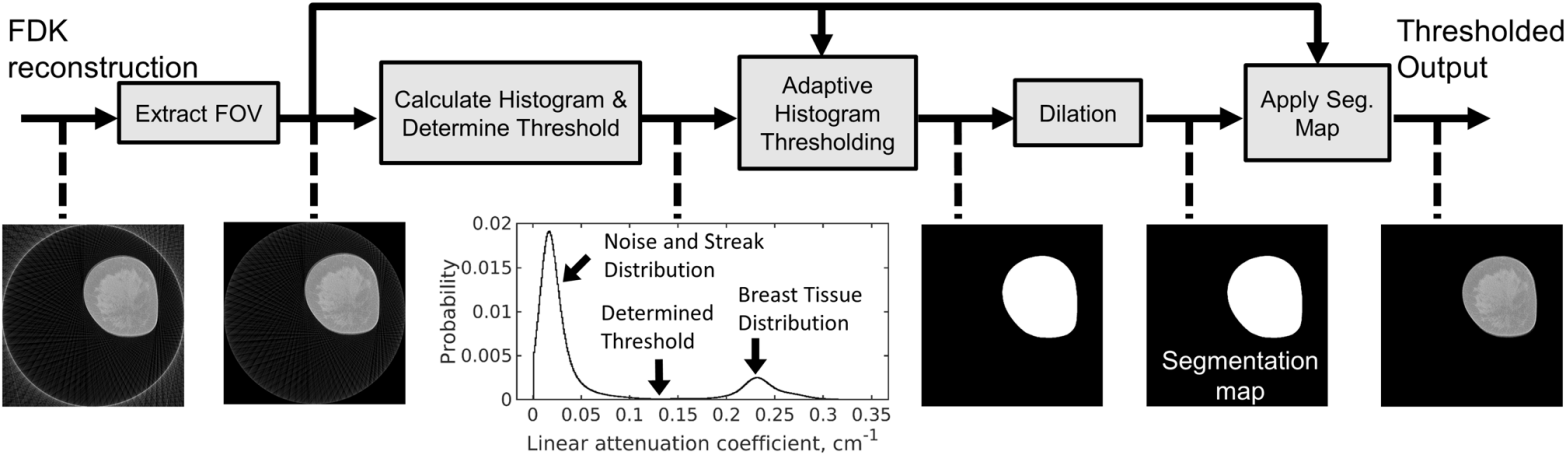
The masking procedure. Circular Field of View (FOV) of the FDK reconstruction is extracted to remove out-of-FOV artifacts. Typically, streaks and breast tissue are well separated in the histogram of linear attenuation coefficients. Based on the histogram, an adaptive thresholding algorithm that selects the bin center with lowest bin counts as the hard threshold is used to generate the segmentation map and the thresholded output. The images and plots linked by dashed line show the intermediate outputs of the entire processing pipeline.

Figure 7a illustrates the proposed training procedure for $Z=3$. The first step in processing is a masking procedure to remove the background regions in each slice. Figure 8 illustrates this masking process for an individual image slice. In this process, masking was performed to remove the artifacts outside of the circular Field of View (FOV). The image data within the circular FOV across all slices were used to create a histogram of linear attenuation coefficients for the entire volume. Based on the observation that the background noise and undersampling artifacts (streaks) are well separated from the breast tissue in this histogram, we selected the bin center with the lowest bin count as the hard threshold and created segmentation maps that identify the breast tissue in each slice. We further dilated the segmentation maps using a flat disk-shaped structuring element with a radius of 2 pixels. Segmentation maps created from the input slices were shared with the corresponding target slices as shown in Fig. 7a. Training is performed using patch pairs extracted from the input and target volumes. Selection of training samples is a well-studied area in machine learning literature and numerous methods have been proposed to reduce bias through training sample selection^68–70^. Inspired by these techniques, patches that contain more than $50\%$ foreground pixels were selected as training samples. This patch extraction process is referred to as tissue-of-interest (TOI) selection.

The network testing phase is illustrated in Fig. 7b. Since the proposed network reconstructs multiple slices simultaneously, a target slice (indicated by dotted yellow bounding box) is reconstructed multiple times in different slice contexts (indicated by red, green, and blue bounding boxes). In this illustration, 5 adjacent slices were first preprocessed using the same masking procedure as the training phase. Using a sliding window of size 3 and stride of size 1, the target slice is processed three times by the network. The three reconstructions are then combined using an ensemble strategy. In summary, for any trained network ${\mathbf {D}}_Z ({{\hat{\mathbf {{w}}}},\cdot} )$ with slice depth *Z*, the ensemble strategy to obtaining the target slice reconstruction ${{\hat{\mathbf {{S}}}}_t}$ can be formulated as

5 $$\begin{aligned} {{\hat{\mathbf {{S}}}}_t} = f(&g_t(\mathbf {D}_Z({\hat{\mathbf {{w}}}}, \mathbf {S}_{t-Z+1}, \mathbf {S}_{t-Z+2}, \cdots , \mathbf {S}_t)),\\&g_t(\mathbf {D}_Z({\hat {\mathbf{w}}}, {\mathbf {S}}_{t-Z+2}, {\mathbf {S}}_{t-Z+3}, \cdots , {\mathbf {S}}_{t+1})),\\&\cdots ,\\&g_t({\mathbf {D}}_Z({\hat{\mathbf{w}}}, {\mathbf {S}}_{t}, {\mathbf {S}}_{t+1}, \cdots , {\mathbf {S}}_{t+Z-1}))) \end{aligned}$$

where *f* denotes the ensemble function, $g_t$ only retains the reconstruction of the target slice *t*, and ${\mathbf {S}}_i$ denotes the slice *i* of the input. In our experiment, we found evenly averaging is a simple yet effective ensemble approach. We replicate border slices to handle slices at edges.

### Network architecture

**Figure 9:**
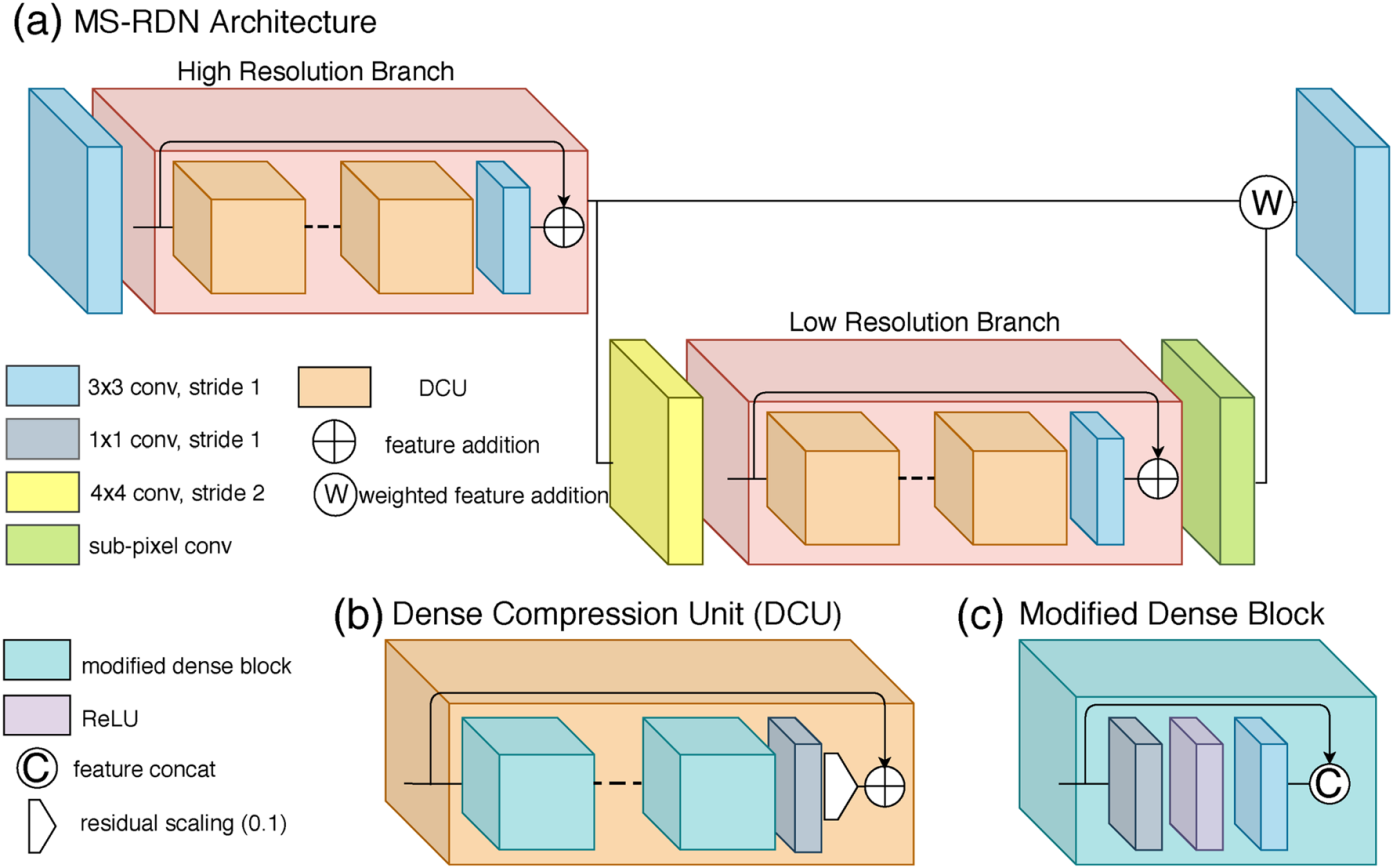
The architecture of multi-slice residual and dense network (MS-RDN). (**a**) Overall layouts; (**b**) the detailed layouts of dense compression unit (DCU); (**c**) the detailed layouts of modified dense block.

The proposed MS-RDN architecture is shown in Fig. 9a. Multi-slice inputs are first processed by a shared 2D convolutional layer. The resulting 3D spatial features are then consecutively propagated through the high resolution and low resolution feature branches. Learned high resolution and low resolution features are summed using a trainable weighting factor. In the end, the output convolutional layer reconstructs multi-slice outputs from the fused feature maps. Inspired by Ledig et al.^44^, our feature branch is sequentially composed of multiple dense compression units (DCUs)^33^, a $3\times 3$ convolutional layer and a skip connection. As shown in Fig. 9b, the DCU consists of stacked densely connected blocks, a $1\times 1$ convolutional layer, a residual scaling (0.1) and a local skip connection. The $1\times 1$ convolutional layer compresses accumulated features to the same number of input features, which enables the residual connection within the dense structure. The constant scaling stabilizes network training, when the number of filters is high^34,71^. The DCU structure efficiently merges local feature information and periodically breaks dense connections to improve back projection of gradients^33^. Figure 9c details the layout of modified dense block, where all batch normalization layers are removed compared to the original DenseNet configuration^32^.

### Network evaluation

To demonstrate the superiority of multi-slice training, we first trained multiple MS-RDNs with the same configurations except for the number of adjacent slices, i.e., $Z=1,3,5,7,9$, respectively. Note that when $Z=1$, MS-RDN reduces to the single slice network, i.e. 2D network.

Our MS-RDN was compared with the residual encoder–decoder convolutional neural network (RED-CNN)^26^ designed for low dose CT image reconstruction. We followed the implementation of RED-CNN from https://github.com/SSinyu/RED_CNN and adopted the suggested network parameters (for example, convolutional kernel size is set to 5). Note that unlike our proposed deep learning reconstruction framework, RED-CNN^26^ was trained with *randomly extracted single-slice patches*. We therefore applied the TOI selection and multi-slice training scheme to the RED-CNN architecture for comparison.

Nine randomly selected test subjects were evenly grouped by the size of breast. To reduce the impact of breast size or slice location, we always select a constant number of measurement samples within the breast for quantitative analysis. The network reconstructions were evaluated with normalized mean square error (NMSE), bias, peak signal-to-noise ratio (PSNR), and Structural Similarity Index Metric (SSIM^42^). The NMSE metric was computed as the ratio of mean square error to mean square of the reference image and converted into decibel (dB), that is

6 $$\begin{aligned} \text {NMSE}({\mathbf {x}},{\mathbf {x}_{ref}})=-10 \times \log _{10}\left( \frac{\left\Vert {\mathbf {x}}-{\mathbf {x}_{ref}}\right\Vert _2^2}{\left\Vert {\mathbf {x}_{ref}}\right\Vert _2^2}\right) . \end{aligned}$$

The bias metric was computed as the mean absolute error. The PSNR metric was computed as the ratio of the maximum pixel intensity ($I_{max}$) squared to mean square error as

7 $$\begin{aligned} \text {PSNR}(\mathbf {x},{\mathbf {x}_{ref}})=10 \times \log _{10}\left( \frac{I_{max}^2}{\left\Vert {\mathbf {x}}-{\mathbf {x}_{ref}}\right\Vert _2^2}\right) . \end{aligned}$$

The SSIM index was computed using the default hyper-parameters except that the dynamic range of pixel values was set to the maximum pixel intensity within the entire dataset. All metrics were calculated in the longitudinal direction as the representation.

The fast, iterative, tv-regularized, statistical reconstruction technique (FIRST^22^) was also used for sparse-view image reconstruction. This algorithm is an ultra-fast variant of the adaptive steepest descent-projection on to convex sets (ASD-POCS^72^) and has been shown to suppress additional artifacts on the periphery of the object. The performance of FIRST was compared to MS-RDN using one small-size breast, one medium-size breast, and one large-size breast.

### Implementation

We construct our MS-RDN with a high resolution branch and a low resolution branch, where each branch consists of 9 DCUs and each DCU is composed of 8 modified dense blocks. The initial number of features is set to 64 with a growth-rate of 32. To evaluate the impact of network depth on RED-CNN performance, we implemented RED-CNN with 10, 22, and 42 convolutional layers. Note that the 10-layer architecture corresponds to what was proposed in the RED-CNN paper^26^ and the 42-layer RED-CNN with $Z=5$ has roughly the same number of trainable parameters (9,243,941) as our MS-RDN with $Z=5$ (9,237,126). In line with observations made in earlier studies^26,73^, we determined that deeper RED-CNNs perform roughly the same as the 10-layer RED-CNN in our application (see Supplementary Fig. S1). Thus, we used the 10-layer RED-CNN for its computational simplicity.

All models were optimized using ADAM with its standard settings $(\beta _1= 0.9,\, \beta _2= 0.999,\, \text {and} \, \epsilon =10^{-8})$ for 100 epochs. Each mini-batch consists of 8 training samples with patch size $128 \times 128 \times Z$, and was normalized by the mean and standard deviation of the entire training data. All networks were trained with $\ell _1$ loss. The learning rate was initially set to $1\times 10^{-4}$ and halved every $2\times 10^5$ mini-batch updates. The single slice network was trained from scratch and used as a pre-trained model for other multi-slice networks. To fine-tune on the pre-trained single slice network, we replicated the single channel weights along the channel dimension at the input and output convolutional layers, respectively^74^. Pre-training, as an approach to initializing network weights, has been shown to improve training stability of larger networks^27,74^. In contrast, we found that further training of the single-slice network does not lead to considerable improvements (see Supplementary Fig. S2). The model with the best validation loss was evaluated at inference time.

Our MS-RDN was implemented in PyTorch^75^ with CUDA backend and CUDNN support, and trained on a NVIDIA Quadro P6000 GPU. The network took about 60 hours on average for 100 epochs training. The FDK and FIRST algorithms were implemented in MATLAB with GPU acceleration. Ram-Lak filter was used for the FDK algorithm and FDK reconstructions were used as the initialization of the FIRST algorithm. Other standard hyperparameters of FIRST were: $\beta =1$, $\beta _{\text {residual}}=0.995$, $\alpha =0.001$, $\alpha _{\text {residual}}=0.95$, $r_{\text {max}}=0.95$, 100 total iterations, and 30 Total Variation iterations. On average, MS-RDN, RED-CNN, FDK, and FIRST require about 2.3 s, 1.2 s, 0.01 s and 3.1 s per slice (1024$\times$1024 matrix size), respectively, on a single NVIDIA Quadro P6000 GPU. Note that MS-RDN and RED-CNN are able to reconstruct breast images in a slice-by-slice manner, whereas FDK and FIRST reconstruct the entire breast volume simultaneously. MS-RDN, RED-CNN, FDK, and FIRST require about 9.0 GB, 2.4 GB, 2.5 GB, and 6.3 GB GPU memory, respectively.

### Statistical analysis

Generalized linear models (repeated measures analysis of variance) were used to test if the metric (NMSE, bias, PSNR, and SSIM) differed between the reconstructions, as the same set of test cases were reconstructed using different methods. Effects associated with $P<0.05$ were considered statistically significant. If the generalized linear model showed significant difference, then follow-up paired t-tests were performed to determine (i) if the metric differed between TOI and non-TOI strategies for MS-RDN and RED-CNN; (ii) if the metric differed between $Z=1$ and $Z=5$ for MS-RDN and RED-CNN; and (iii) if MS-RDN differed from RED-CNN for the TOI strategy when $Z=1$ and $Z=5$. For each metric, this results in a total of 7 comparisons. Hence, Bonferroni-adjusted alpha of 0.007 was considered statistically significant for these pairwise comparisons. The data analysis for this paper was generated using SAS software, Version 9.4 of the SAS System for Windows.

## Supplementary information

Supplementary Information.
