# Supplementary material for: A residual dense network assisted sparse view reconstruction for breast computed tomography
Source: Sci Rep. 2020 Dec 3;10:21111. doi: 10.1038/s41598-020-77923-0 (PMC7713379; doi:10.1038/s41598-020-77923-0)
Supplement: Supplementary file 1 — Supplementary Information. [file 41598_2020_77923_MOESM1_ESM.docx]

A Residual Dense Network Assisted Sparse View Reconstruction for Breast Computed Tomography

Zhiyang Fu^1,2^, Hsin Wu Tseng ^1^, Srinivasan Vedantham ^1,3^, Andrew Karellas ^1^, and Ali Bilgin ^1,2,3*^

^1^ Department of Medical Imaging, University of Arizona, Tucson, Arizona, United States

^2^ Department of Electrical and Computer Engineering, University of Arizona, Tucson, Arizona, United States

^3^ Department of Biomedical Engineering, University of Arizona, Tucson, Arizona, United States

^*^ bilgin@email.arizona.edu


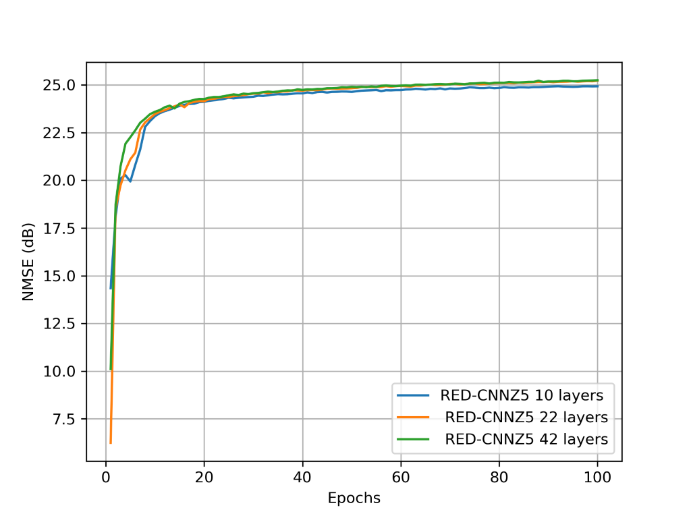

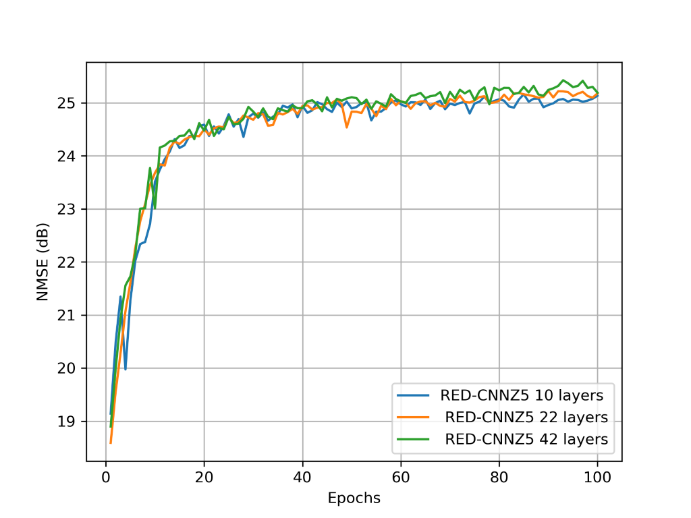


(a) Training (b) Validation

Supplementary Figure S1: (a) Training (b) Validation NMSE evolution for RED-CNNZ5 with 10, 22, and 42 convolutional layers. All three networks converged to roughly the same NMSE during training or validation.


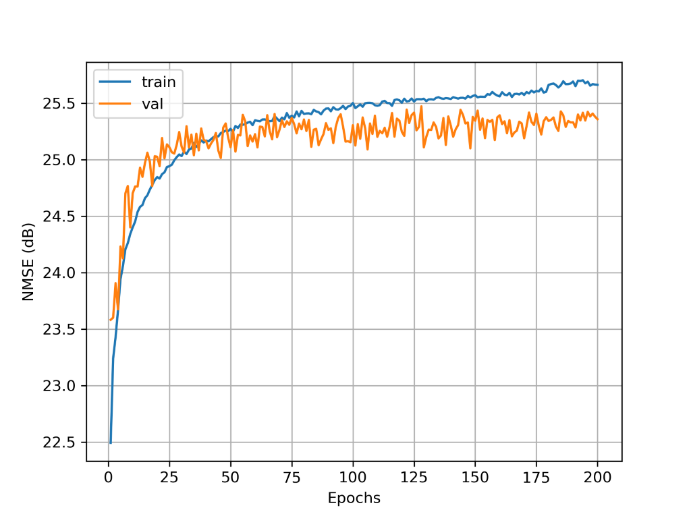

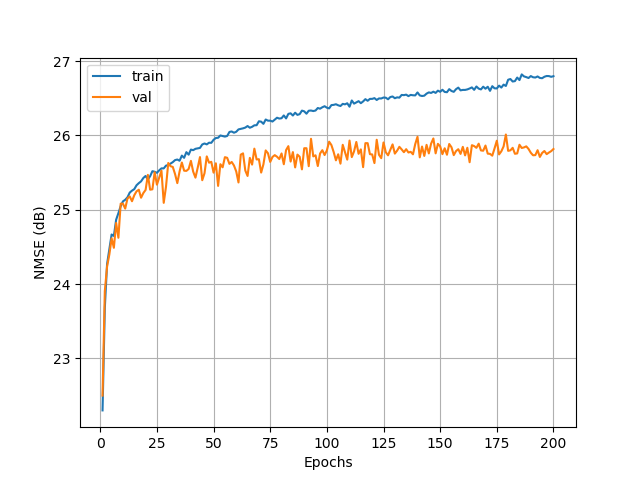


(a) RED-CNNZ1 (b) MS-RDNZ1

Supplementary Figure S2: The training and validation curves for (a) RED-CNNZ1 and (b) MS-RDNZ1 over 200 epochs. The training NMSEs in (a) and (b) continue to improve after 100 epochs whereas the validation NMSEs are saturated at around 100 epochs and do not show any substantial improvement afterwards.
